# Dr. Elizabeth Corwin

**Published:** 1914-12

**Authors:** 


**Dr. Elizabeth Corwin,** N. Y. Medical College and Hospital for Women, formerly of Binghamton, died at her home in Battle Creek, August 18, aged 62.

